# Coastal Dune Vegetation Dynamism and Anthropogenic-Induced Transitions in the Mexican Caribbean during the Last Decade

**DOI:** 10.3390/plants13131734

**Published:** 2024-06-23

**Authors:** Eloy Gayosso-Soto, Sergio Cohuo, Joan Alberto Sánchez-Sánchez, Carmen Amelia Villegas-Sánchez, José Manuel Castro-Pérez, Leopoldo Querubín Cutz-Pool, Laura Macario-González

**Affiliations:** 1 Tecnológico Nacional de México/IT de Chetumal, Av. Insurgentes 330, Chetumal 77013, Quintana Roo, Mexico; mere10.eloy@gmail.com (E.G.-S.); carmen.vs@chetumal.tecnm.mx (C.A.V.-S.); jose.cp@chetumal.tecnm.mx (J.M.C.-P.); leopoldo.cp@chetumal.tecnm.mx (L.Q.C.-P.); 2 Department of Sustainability Sciences, El Colegio de la Frontera Sur, Avenida Centenario Km 5.5, Chetumal 77014, Quintana Roo, Mexico; jasanchez@mail.ecosur.mx; 3 Tecnológico Nacional de México/IT de la Zona Maya, Carretera Chetumal-Escárcega Km 21.5, Ejido Juan Sarabia 77965, Quintana Roo, Mexico; laura.mg@zonamaya.tecnm.mx

**Keywords:** Mexican Caribbean, coastal dune vegetation, Landsat 7 remote sensors, SAVI index

## Abstract

In the Mexican Caribbean, environmental changes, hydrometeorological events, and anthropogenic activities promote dynamism in the coastal vegetation cover associated with the dune; however, their pace and magnitude remain uncertain. Using Landsat 7 imagery, spatial and temporal changes in coastal dune vegetation were estimated for the 2011–2020 period in the Sian Ka’an Biosphere Reserve. The SAVI index revealed cover changes at different magnitudes and paces at the biannual, seasonal, and monthly timeframes. Climatic seasons had a significant influence on vegetation cover, with increases in cover during northerlies (SAVI: *p* = 0.000), while the topographic profile of the dune was relevant for structure. Distance-based multiple regressions and redundancy analysis showed that temperature had a significant effect (*p* < 0.05) on SAVI patterns, whereas precipitation showed little influence (*p* > 0.05). The Mann–Kendall tendency test indicated high dynamism in vegetation loss and recovery with no defined patterns, mostly associated with anthropogenic disturbance. High-density vegetation such as mangroves, palm trees, and shrubs was the most drastically affected, although a reduction in bare soil was also recorded. This study demonstrated that hydrometeorological events and climate variability in the long term have little influence on vegetation dynamism. Lastly, it was observed that anthropogenic activities promoted vegetation loss and transitions; however, the latter were also linked to recoveries in areas with pristine environments, relevant for tourism.

## 1. Introduction

In Mexico, the area occupied by the coastal dunes is ~808,711 ha [1]. Nevertheless, it is estimated that about 50% of this area has been anthropogenically modified into agricultural land, urbanized land, or land used for touristic services [1,2,3]. The Mexican Caribbean coast represents 1.5% of the Mexican coastal area and is characterized by a discontinuous dune system, mostly of the frontal type, covering ~12,278 ha [1]. The coastal dunes of the Caribbean are characterized by their vegetation, which is structured by dune geomorphology. Pioneer vegetation present in the mobile zone includes *Canavalia rosea* (Sw.), *Ipomoea pes-caprae* (L.R.Br.), *Sesuvium portulacastrum* (L. L., 1759), *Sporobolus virginicus* (L. Kunth), *Ambrosia hispida* (Pursh), and *Cakile lanceolata* (Willd. O. E. Schulz). On the established dunes, common species are *Suriana maritima* (L.), *Tournefortia gnaphalodes* (L.R.Br. ex Roem. & Schult), *Ernodea littoralis* (Sw.), *Scaevola plumieri* (L. Vahl), *Chrysobalanus icaco* (L.), and *Lantana involucrata* (L.). Lastly, the back-dune is mostly dominated by shrubs such as *Thrinax radiata* (Lodd. ex Schult. & Schult.f.), *Coccoloba uvifera* (L.), *Cordia sebestena* (L.), *Bursera simaruba* (L. Sarg.), *Metopium brownei* (Jacq. Urb.), and *Cocos nucifera* (L.). All these species are key in the formation processes, establishment, and stabilization of the coastal dunes in the Caribbean [1,4,5,6,7,8,9].

Given the economic relevance of tourism in the Mexican Caribbean, the dune system has been altered to different degrees, including incipient modifications such as the creation of pedestrian pathways up to the complete removal of these ecosystems [10]. In the central–northern zone of the Mexican Caribbean, the rapid urbanization of coastal cities such as Cancún, Playa del Carmen, and Tulum (>1 million inhabitants) and the development of beachfront touristic complexes between cities has resulted in the removal of extensive areas of coastal dunes, as well as their connectivity with other ecosystems such as mangroves, reefs, cenotes, and forests [10]. By 2016, only 34% of the total surface of the Mexican Caribbean coastal dune was estimated to be in its natural state; 17% was fragmented by roads and paths, 26% disturbed for different uses (farming, human settlements), and 23% replaced for exclusive urban and touristic use [1,11]. In areas with anthropogenic influence, vegetation transitions have also been observed, especially the replacement of native species by exotic species. This occurs accidentally or deliberately through the introduction of crops and ornamentals, and as a result, they are broadly distributed in the coastal zones [1,6,12,13,14,15]. The most extensively distributed exotic species in the Mexican Caribbean are *C. nucifera*, *Scaevola taccada* (Gaertn. Roxb.), and *Casuarina equisetifolia* (L.), all directly competing with native species of the mobile and established dune zones [15,16]. Furthermore, the Mexican Caribbean is a climatically sensitive region that is yearly, directly or indirectly, altered by hydrometeorological events such as tropical storms and hurricanes [10,16] with the potential of modifying the geomorphology and vegetation of coastal dunes [17]. Reductions in precipitation and increases in temperature as a result of global climate change in this region are additional drivers of vegetation change in coastal dunes [17,18,19]. Long-term effects of anthropogenic activities and natural dynamics on the coastal dune and its vegetation are difficult to estimate in the Caribbean region [10,11,20], especially in Mexico, where pristine environments that may serve as references are nonexistent and historical continuous records of vegetation change are limited.

The Sian Ka’an Biosphere Reserve (SKBR) in the central portion of the Mexican Caribbean was declared a natural reserve in 1986 to protect approximately ~80km of beachfront and about 1600 ha of coastal dune vegetation [21]. The Territorial Ecological Planning Program of the coastal zone of the SKBR and the Management Program of the Protected Natural Area with the status of Sian Ka’an Biosphere Reserve are the legal instruments that plan and organize the reserve through environmental policies, ecological criteria, and administrative rules for the protection, preservation, management, and sustainable use of its resources [21,22,23]. However, these same instruments allow land use changes to some extent under an economically sustainable development model through land privatization, authorization of touristic activities, and infrastructure [24,25]. The SKBR is suitable for evaluating long-term natural coastal vegetation dynamism and anthropogenic influence in the Mexican Caribbean, which is critical for establishing an ecologically referenced framework for the conservation of coastal dune vegetation at a regional level.

Aerial photogrammetry, remote sensing, and geographic information systems have been successfully applied to evaluate changes in vegetation in tropical regions [26,27,28,29]. To date, 12 types of land uses have been recognized in the coastal dunes of the Mexican Caribbean. This was possible through satellite imagery, combining the Spot 6 sensor and Rapideye during the 1986–2015 period [7]. Some of these land uses are associated with coastal vegetation such as *Ambrosia hispida* community, *Ernodea littoralis* community, other frontal dune communities, chital (*Thrinax radiata*) forest, mixed arboreal–shrub communities, mangrove and other wetlands, disturbed mangroves, and coconut plantations. However, four other land uses, including lagoons and estuaries, sandy beaches, rocky beaches, and roads and buildings, are not directly related to vegetation [7]. Moreover, it has also been recognized through satellite imagery that the topographic profile of the dune does not influence the distribution and organization of its floristic community, and therefore, the drivers of vegetation change in the short and long term remain uncertain [5,6,7]. In this study, we aim to contribute to answering the following research questions. 1) What was the spatial distribution of vegetation cover in the coastal dunes of the central Mexican Caribbean during the 2011–2020 period? 2) What were the factors (anthropogenic vs. climate) that influenced the changes in the structure of vegetation cover?

## 2. Results

### 2.1. Spatial and Temporal Vegetation Cover Density Variability in the Coastal Dune of SKBR during 2011–2020

The Spearman non-parametric test revealed a strong correlation between the SAVI and NDVI performance by area and year (NZ: r = 0.985; *p* = 0.000; SZ: r = 0.995; *p* = 0.000). However, because SAVI better discriminates the soil factor in coastal areas, the performance of the SAVI index was used to analyze the last decade’s variability in vegetation cover.

In the northern zone, the estimated biannual maximum value of SAVI was 0.64 and the minimum value was −0.77. Mean SAVI values varied little among biannual layers. During 2011–2012, for example, the mean and median values were 0.39 and 0.44, respectively, while for the 2013–2014 period, the mean value was 0.46 and the median value was 0.40.

In the southern zone, biannual SAVI maximum and minimum values were 0.66 and −0.76, respectively. Mean values among biannual layers slightly varied. During the 2011–2012 period, the mean value was 0.32 and the median value was 0.35, whereas for 2013–2014, the mean and median values were 0.32 and 0.34, respectively. Moreover, the layer for the 2019–2020 period recorded the maximum mean and median values of 0.35 and 0.35, respectively.

**Figure 1:**
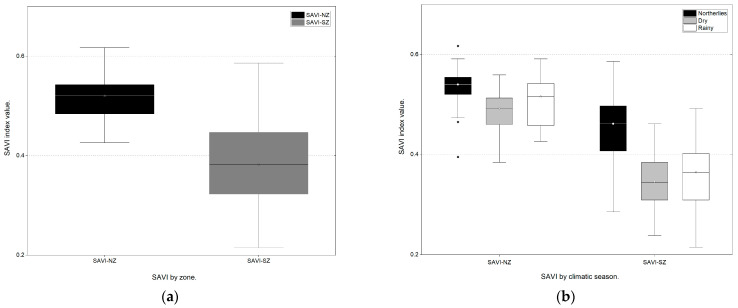
Box and whisker plots for the SAVI values by (**a**) zone and (**b**) climatic season. Solid horizontal lines represent the median of each group. Extreme outliers are represented by black dots. SAVI showed higher values and was more homogeneous in the NZ than in the SZ. SAVI showed higher values during the northerlies than during other climatic seasons.

For the comparison of SAVI values by zone and climatic season, the initial K-S test revealed that both the NZ and the SZ did not present a normal distribution (*p* = 0.000; *p* = 0.011; *p* < 0.05). Additionally, the UMW test showed that the vegetation cover in the NZ and SZ was significantly different (*p* = 0.000; *p* < 0.05) during the study period (Figure 1a).

Similarly, significant differences were found between climatic seasons (northerlies, dry and rainy seasons; K-W: *p* = 0.000; *p* = 0.000; *p* < 0.05) in both zones. The Bonferroni multiple correction comparisons test indicated that the vegetation covers during dry and rainy seasons (*p* = 0.255; *p* = 1.0; *p* > 0.05) were not significantly different; however, a significant difference in vegetation cover was observed during northerlies (Figure 1b).

### 2.2. Vegetation Cover Density Trend with SAVI

**Figure 2:**
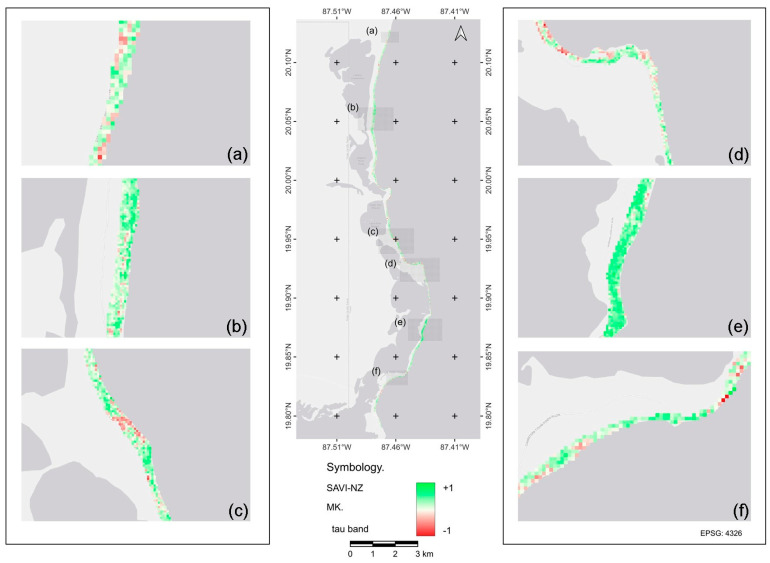
Distribution of the Mann–Kendall trend in the northern zone (NZ) of the Sian Ka’an Biosphere Reserve estimated from SAVI for the period 2011–2020. Maps (**a**–**f**) represent sections of the NZ overall tendency map.

**Figure 3:**
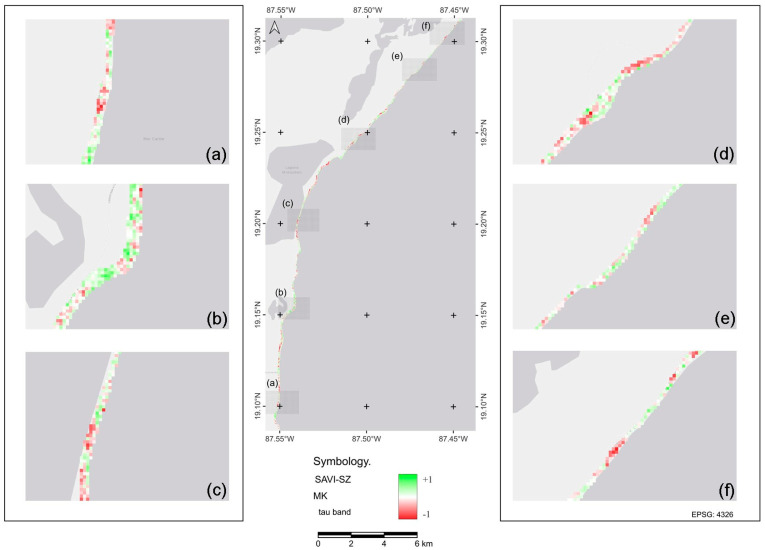
Distribution of the Mann–Kendall trend in the southern zone (SZ) of the Sian Ka’an Biosphere reserve estimated from SAVI for the period 2011–2020. Maps (**a**–**f**) represent a section of the overall tendency map of the SZ.

The Mann–Kendall test showed trends in vegetation change (*p* < 0.0003; *p* < 0. 0006; *p* < 0.05) for both the NZ and SZ. At the local level (pixel extent, representing areas of 30 x 30 m), we detected zones with continuous positive trends, representing different vegetation recovery processes with permanent or enhanced photosynthetic activity. These trends were more frequently observed in the NZ. Negative trends showed areas with decreased photosynthetic activity and thus a reduction in vegetation cover (Figure 2 and Figure 3). These trends were more frequent in the SZ. Areas with a constant neutral tendency and more evident stable photosynthetic activity were more frequently observed in the NZ (Figure 2a,d,f).

### 2.3. Vegetation Cover Density Evolution in the Northern and Southern Zone of the SKBR

**Figure 4:**
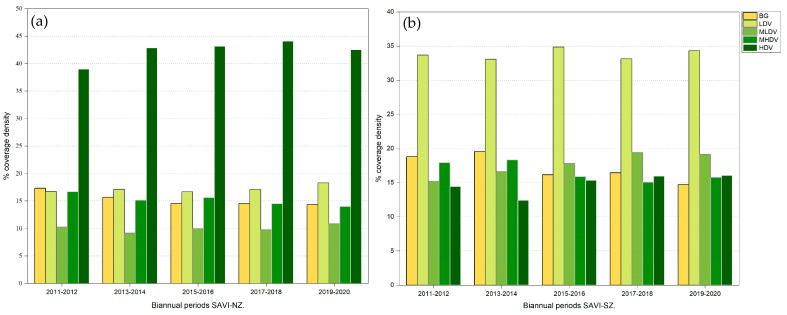
Percentage of vegetation cover density for biannual periods for the coastal dune ecosystem in the (**a**) northern zone and (**b**) southern zone of the Sian Ka’an Biosphere Reserve. Abbreviations are as follows: Bare Soil (BG); Low-Density Vegetation (LDV); Medium–Low-Density Vegetation (MLDV); Medium–High-Density Vegetation (MHDV); and High-Density Vegetation (HDV).

Vegetation cover density changed over time and space in the NZ and was highly variable with respect to the different categories (Table 1). The highest density of vegetation cover ranged between 0.45 and 0.64. These values corresponded to the High-Density Vegetation category (stratum: arboreal), representing 57.52% of the total surface area. The most relevant changes were observed within the spatial cover of the Low-Density Vegetation category (stratum: herbaceous and creeping vegetation), which increased from 16.74% during 2011–2012 to 18.29% during 2019–2020 (Figure 4a). The High-Density Vegetation category (stratum: arboreal) initially increased from 38.99% during 2011–2012 to 44.07% in the 2017–2018 period. However, during 2019–2020, this value decreased to 42.51% (Figure 4a). Medium–High-Density Vegetation (stratum: shrubby–arboreal) exhibited a cover of 16.68% for the 2011–2012 period, which decreased to 13.98% during the 2019–2020 period. Medium–Low-Density Vegetation (stratum: shrubby) showed a cover of 10.26% during 2011–2012, but decreased, ranging from 9.17% to 9.97 during biannuals 2013–2014, 2015–2016, 2017–2018, and further showing a recovery to 10.85% during 2019–2020 (Figure 4a). Regarding the spatial cover of the Bare Soil category (sand), this reduced gradually from 17.33% to 14.36% during the 2019–2020 period (Figure 4a).

According to the SAVI category, in the SZ, the highest density of vegetation cover ranged from 0.45 to 0.66, which corresponded to High-Density Vegetation (stratum: arboreal) and represented 25.70% of the total surface area. The Low-Density Vegetation category (stratum: herbaceous and creeping vegetation) increased from 33.08% during 2013–2014 to 34.85% in the 2015–2016 period and remained constant during the other periods (Figure 4b). Medium–Low-Density Vegetation (stratum: shrubby) ranged from 15.17% (2011–2012) to 19.41% (2017–2018) (Figure 4b). Medium–High-Density Vegetation (stratum: shrubby–arboreal) showed cover reductions from 18.33% (2013–2014) to 15.06% (2017–2018) (Figure 4b). High-Density Vegetation (stratum: arboreal) presented a cover of 14.42% for the period 2011–2012, with a slight decrease to 12.42% during 2013–2014, a constant cover recovery in the following periods, and up to 16.06% in the last period (2019–2020) (Figure 4b). The Bare Soil (sand) showed a spatial cover of 18.8% for the period 2011–2012, decreasing to 14.71% during 2019–2020 (Figure 4b).

### 2.4. Biannual Changes in Vegetation Cover Density in the Northern and Southern Zone of the SKBR

**Figure 5:**
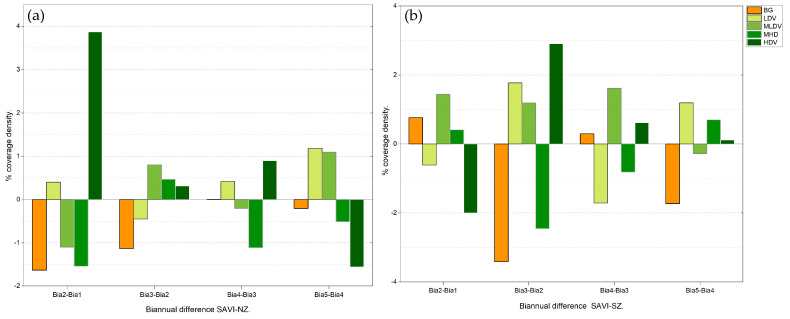
Biannual differences in vegetation cover density (expressed in percentage) as per the SAVI index for the coastal dune ecosystem in the (**a**) northern and (**b**) southern zones of the SKBR. Abbreviations for the x-axis are as follows: Bia: biannual periods, Bia1: 2011–2012; Bia2: 2013–2014; Bia3: 2015–2016; Bia4: 2017–2018; Bia5: 2019–2020. Abbreviations in the symbology are the same as in Figure 4.

The High-Density Vegetation category (stratum: arboreal) showed cover variations of 3.87%, 0.32%, and 0.90%. Cover losses were observed from the 2017–2018 period to the 2019–2020 period (Figure 5a), whereas the most relevant recovery occurred during the 2011–2012 and 2013–2014 biannual periods. In the NZ, the Bare Soil (sand) category showed a loss of cover area (−0.2 to −1.63%) throughout the evaluated period. Similarly, the area covered by Low-Density Vegetation (stratum: herbaceous and creeping vegetation) and Medium-Low Density Vegetation (stratum: shrubby) increased by 1.18% and 1.09% during the period 2017–2018 to 2019–2020, respectively (Figure 5a).

During the periods 2013–2014 and 2015–2016, the High-Density Vegetation category (stratum: arboreal) increased its cover to 2.9% but subsequently decreased to −2%, 0.62%, and 0.12% during the 2011–2012, 2017–2018, and 2019–2020 periods, respectively (Figure 5b). Bare Soil (sand) showed an overall decreasing tendency (−1.73 to −3.41%), denoting an increase in vegetation in coastal dunes in the SZ. The Low-Density Vegetation (stratum: herbaceous and creeping vegetation) and Medium–Low-Density Vegetation (stratum: shrubby) presented fluctuating cover areas, with 1.77% as the highest observed value (2013–2014 to 2015–2016), as well as 1.20% (2017–2018 to 2019–2020), 1.44% (2011–2012 to 2013–2014), 1.19% (2013–2014 to 2015–2016), and 1.61% (2015–2016 to 2017–2018) (Figure 5b).

### 2.5. SAVI Relationship and Environmental Climate Variables (CVs)

**Figure 6:**
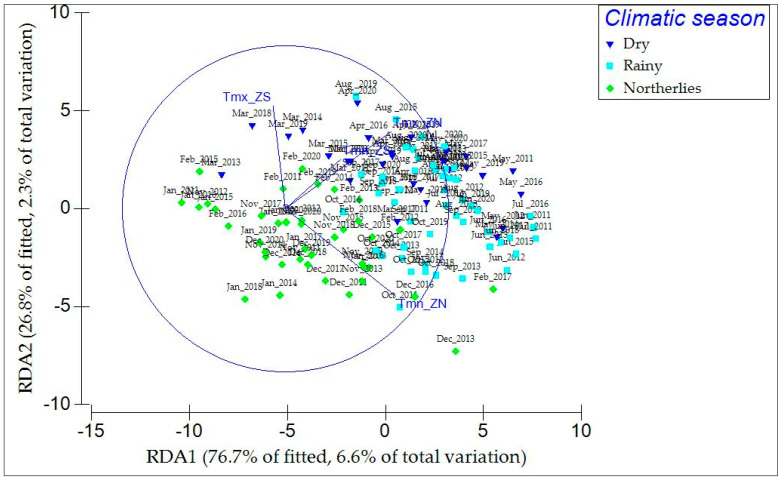
Spatial representation of the variation exhibited by SAVI values in the NZ and SZ by the different climatic seasons (northerlies, dry, and rainy). The most significant environmental climatic variables were Tmx: maximum temperature (°C), Tmn: minimum temperature (°C), and Prep: precipitation (mm).

The DistLM test showed that the climatic variables, maximum temperature (*p* < 0.0022; *p* < 0.0051) and minimum temperature (*p* < 0.0007; *p* < 0.0006), had a significant effect (*p* < 0.05) on the distribution of SAVI values for the 2011–2020 period in both the NZ and SZ (Tables S1 and S2). The dbRDA routine on its first two axes captured >76.6% of the variability in the fitted model and >6.6% of the total variation in the data. The axes suggest that the results obtained from the SAVI index were mainly influenced by temperature changes that coincided with the dry and rainy seasons. In contrast, precipitation (Prep mm: *p* > 0.0889; *p* > 0.1664) did not exhibit any significant effect (*p* > 0.05) on the distribution of the data obtained from the SAVI analysis (Figure 6).

## 3. Discussion

### 3.1. Spatial and Temporal Vegetation Cover Density Variability in the Coastal Dune of RBSK

In this study, SAVI values ranged between 0.39 and 0.64, similar to those recorded in other Mexican regions, such as on the coasts of the Pacific Ocean. Rodríguez-Moreno and Bullock [30] reported similar mean SAVI values that ranged between ~0.26 and 0.65 for coastal vegetation in the Baja Californian Peninsula using the Landsat 5 TM sensor (30 m resolution). Additionally, Ceceña-Sanchez et al. [31] reported mean SAVI values ranging from 0.33 to 1.00 for the coastal vegetation in Guadalupe Island for forests, palm groves, and shrubby scrub using QuickBird and WorldView-2 (resolutions of 2.4 m and 2 m). Therefore, values obtained in this work can be considered within the range of values previously reported for coastal vegetation in the region.

The overall results of SAVI suggested dynamism in vegetation cover during the last decade in the SKBR; however, it was observed that the magnitude of such dynamism presented seasonal fluctuations. In the biannual spatial analysis, SAVI patterns narrowly fluctuated between 0.39 and 0.64, suggesting relatively stable coastal vegetation conditions. The SAVI patterns, however, were significantly different between the NZ and SZ, demonstrating that the drivers of these changes, such as the topographic profiles of the dunes, may have different implications at a local scale. In this context, the dune system of the NZ has an extension of 4–19 m with steep slopes, whereas in the SZ, the system has an extension of 4–11 m with more gentle slopes [5,6,7]. The topographic profile is strongly related to the arrival, establishment, and removal of plant cover in the dune system [1,12,13]. In the NZ, for example, the topographic profile favors the establishment of mangrove vegetation in areas close to the sea that are in association with flora, characterized by long-term permanence and broad cover, such as *T. radiata*, *A. hispida*, *E. littoralis*, *C*, *uvifera*, *S. maritima*, *T. gnaphalodes*, *C. nucifera*, and other herbaceous plants such as *S. portulacastrum*, *I. pes capre*, *S. virginicus*, and *C. lanceolata* [1,4,5,7]. Therefore, from a biannual perspective, the vegetation dynamics are less perceivable since the plant community will tend to recover in the event of short-term disturbances.

The seasonal analysis (northerlies, rainy, and dry seasons) showed a higher association with the dynamism of vegetation cover than the biannual context. During the northerlies, SAVI displayed the highest values, which were significantly different compared to those of the rainy and dry seasons, where the vegetation had remained more stable. During the northerlies, climatic conditions may be variable for both temperature and precipitation. During the ten-year period evaluated in this study, temperature values tended to be relatively lower (17.4 °C to 23.1 °C) than those of other climatic seasons (27.80 °C to 32 °C), agreeing with TerraClimate data. Precipitation values varied through time, influenced by both polar airmass movement and hydrometeorological phenomena such as hurricanes and tropical storms. According to NOAA [32], six hydrometeorological phenomena were recorded during the evaluated period, with indirect impacts on the study area. Nevertheless, erosive effects and changes to vegetation were recorded at the regional level [7,33]. The combined effects of temperature (Tmx, Tmn; Figure 6) and sparsely distributed rains seemed to have a positive influence on the development of and increase in vegetation cover, consequently reflected in the SAVI values obtained [34,35]. De Almeida et al. [36], D’alessandro et al. [37], and Hogan et al. [19] have previously indicated that physical factors in the dune ecosystem, such as sediment hauling and transport and the interconnectivity with the adjacent reef ecosystem, are all affected during the northerlies and hurricane events, thus contributing to the dynamism of this ecosystem and its vegetation.

During the dry and rainy seasons, the Bonferroni multiple correction comparison test indicated that the vegetation covers were not significantly different. This is an interesting result, as precipitation is apparently highly contrasting between these seasons. The RDA, in fact, revealed a more significant influence of temperature (maximum and minimum) than precipitation on vegetation. Temperature in the Yucatan Peninsula plays an important role in controlling plant phenology and physiology [38,39], where it has been observed that plant evapotranspiration in tropical dry deciduous forests, driven by solar incidence, is relatively similar between the dry and rainy seasons [40]. Thus, we consider that temperature plays a key role in coastal vegetation dynamism, since prolonged episodes of droughts (when freshwater is unavailable) during the dry season may be endured by plants with a higher tolerance to salinity, thus leading to minimal plant cover disturbance between seasons.

### 3.2. Trends in Vegetation Cover Density in the SKBR

**Figure 7:**
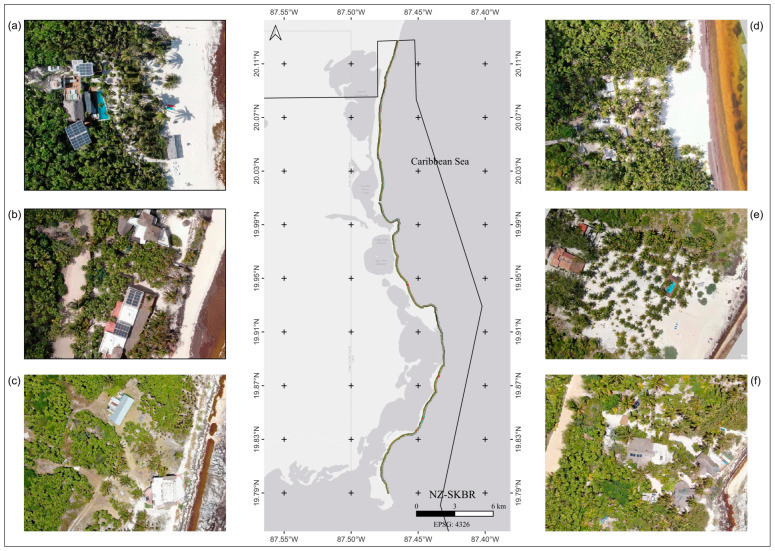
Map showing the northern zone of Sian Ka’an Biosphere Reserve. Drone images (**a**–**f**) showing the anthropogenic impact on the dune ecosystem and its vegetation.

The Mann–Kendall test recorded significant annual trends for vegetation change in the SKBR. The overall trend suggested that while vegetation recovery was evident in the NZ, vegetation loss was observed in the SZ. Figure 4 and Figure 5, however, show that such a tendency was not homogeneous throughout the NZ and SZ, with some well-delimited zones presenting a higher impact, mainly consistent with human settlements. The SEMARNAT-CONAP [41] Mexican agencies dedicated to the protection of the environment estimate that 90% of the surface of the SKBR has been impacted to some degree by anthropogenic activity and that a portion of the native vegetation in the coastal dune has been replaced by coconut trees. By 2015, these agencies estimated that ~54.34 ha of coastal dune had been severely transformed. Elizondo et al. [7] estimated that from 1986 to 2015, ~5.8% of natural vegetation was lost in the coastal dune as a result of new infrastructure such as roads, buildings, and also the cultivation of coconut palm trees. Furthermore, Guimarais et al. [42] found that during 2015–2020, the loss of coastal dune area peaked at ~23.7 ha in the SKBR. Therefore, anthropogenic activity seems to be the main driver of annual changes in vegetation in the coastal dune, as evidenced by the loss of Medium–Low- and Low-Density Vegetation categories, which are mostly affected by settlements and the economic use of coastal areas. Anthropogenic disturbance in the area can be tracked back to the 1930s, with the establishment of coastal communities such as Punta Allen and Punta Herrero, accompanied by the establishment of cattle ranches and grazing activities, fisheries, land use change, land subdivisions, infrastructure, and tourist activities along the coastline (Figure 7) [1,11,25,42,43,44]. Currently, with all the restrictions coming from the designation of the region as a Biosphere Reserve, the distribution and abundance of *C. nucifera* has decreased by more than 80% [5,7,41].

The recovery tendency in the NZ may be explained by the touristic activities in the zone. Historically, the NZ has had an important tourist influx and infrastructure [5,7,25,45] due to its proximity to touristic centers such as Tulum. However, the conservation and restoration of natural environments are currently a priority because of the predominant ecotourism in the area. In this region, social and regional phenomena have been observed in which local communities advance their economies by protecting natural resources even in the absence of legal regulations and laws, with the aim of promoting pristine environments to tourists and recreational activities within these areas. This has been observed as a regional tendency occurring similarly in Belize and Bonaire in the Wider Caribbean [46,47]. As a result, relative protection and conservation seem to promote vegetation recovery from the early and long-term alterations caused by economic activities such as cattle rearing and coconut cultivation.

**Figure 8:**
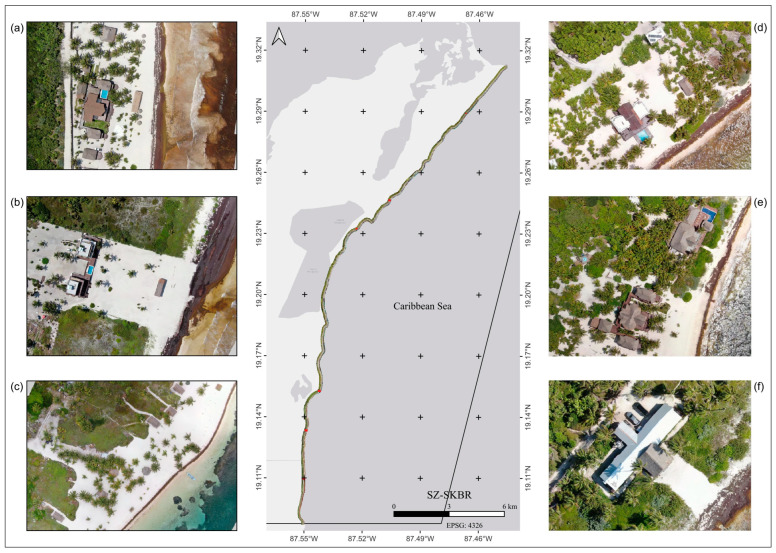
Map showing the southern zone of Sian Ka’an Biosphere Reserve. Drone images (**a**–**f**) showing the anthropogenic impact on the dune ecosystem and its vegetation.

In the SZ, the decrease in vegetation cover can be explained by the predominant activities in the zone, resulting from local land subdivisions. The most common activities in the SZ are the construction of new touristic spaces, cattle farming, and urbanization (Figure 8). The coastal dune in this zone is dominated by shrubby (MLDV) and herbaceous (LDV) vegetation [5,7] that are more sensitive to the effects of climate variability, which may be contributing to the decreasing trend observed in this zone.

### 3.3. Changes in Vegetation Cover Density during the Last Decade in the SKBR

Vegetation density changed drastically over the years and within the assessed zones, illustrating high dynamism and vegetation rearrangements. In the NZ, High-Density Vegetation (stratum: arboreal) and Bare Soil (sand) categories showed cover loss, whereas in the SZ, High-Density Vegetation (stratum: arboreal), Low-Density Vegetation (stratum: herbaceous and creeping), Medium–Low-Density Vegetation (stratum: shrubby–arboreal), and Bare Soil (sand) categories were the most affected. This dynamism can be explained by different factors. For instance, in both regions, broad areas that are in proximity to the coast are dominated by mangroves, and the reflectance in these zones can be influenced by moisture content and the amount of water under the canopy, producing local variability [48,49]. We also assume that vegetation changes could be related to anthropogenic activities and touristic infrastructure in the area (Figure 7 and Figure 8). It was also observed that High-Density Vegetation (stratum: arboreal), mainly composed of mangroves, was affected by up to 4% of the changes between years.

The removal of mangroves is still a common practice in remote regions of the SKBR where environmental surveillance is limited. Landowners progressively remove mangrove and associated species for the construction of pathways first, then houses, and infrastructure [43]. The removal of Medium-Low and Low-Density Vegetation (stratum: herbaceous, creeping, and shrubby) is strongly correlated with erosive effects on the Caribbean coasts and the movement of the sea further inland, as evidenced on the Costa Rican and Curaçao coasts [50,51]. In the Colombian Caribbean, the loss of mangroves is significant; however, their long-term effects are still unknown. In the coastal cities of the Mexican Caribbean, such as Cancun, Cozumel, and Playa del Carmen, erosive effects linked to the removal of vegetation are now a social problem, as some parts of the coastline have retreated after natural disturbances, thus affecting popular beaches. In 2009, 7 million m^3^ of sand fill was necessary after the indirect effects of hurricanes Wilma (2005) and Dean (2007) in the northern Mexican Caribbean [52,53]. In 2020, 1.7 million m^3^ of sand fill was newly required to recover beaches in the same regions [54].

Hogan et al. [19] highlighted that disturbances caused by hurricanes in forest ecosystems (tropical, subtropical, and temperate) induce vegetation dynamism, shaping the structure and composition of species in coastal and continental areas, in addition to altering environmental conditions such as solar radiation, soil moisture, waste deposition, and nutrient cycling [55,56,57,58,59,60,61,62]. This work also established that solar radiation alone has little to no effect on the dynamics of the coastal floristic community, but its effects may be more evident only after canopy and/or vegetation cover loss, causing shifts in species composition from regenerating understory to pioneer species. Vegetation dynamism is therefore induced by natural events (hurricanes and storms) causing shifts in solar radiation and soil humidity [19]. In our study area, climate favors the presence of cloudy skies and cloud shadows, with up to 200 cloudy days per year [41], thus reducing the impact of radiation on the vegetation cover most of the year.

Other vegetation categories, such as Low-Density Vegetation (stratum: herbaceous and creeping) and Medium–Low-Density Vegetation (stratum: shrubby), mainly composed of the herbaceous and shrubby strata in different phases of development, were also observed to be highly dynamic. This type of vegetation is more sensitive to climate variability, which in turn is recognized as the main driver of the distribution, cover, and abundance of plant species [12,35,63,64]. The topographic profile is an additional factor that promotes ecological succession under climate variability and hydrometeorology [2,3,4,5,6]. Some species (*C. rosea*, *I. pes-caprae*, *S. portulacastrum*, *S. virginicus*, *C. lanceolate*, *S. maritima*, *T. gnaphalodes*, *E. littoralis*, *S. plumieri*, *C. icaco*, among others) in this stratum (Table 1) have developed specific adaptations to resist burying, nutrient concentration, saline spray, water scarcity, salinity, and fluctuating temperatures [2,3,7,8,9,65]. According to Hernández-Cordero [66], changes in temperature and precipitation significantly influence the growth of vegetation in the coastal dune. In Mexico, shrubby vegetation, such as *S. maritima*, *T. gnaphalodes*, and *C. icaco*, present on the coastal dune is more tolerant to burial than herbaceous species and other species like *Chamaecrista chamaecristoides* (Collad. Greene *var. chamaecristoides*), with the ability to resist up to three months of drought [6,8,10,67]. Therefore, dry periods in the dunes could be more dynamic, due to the loss of vegetation cover [68]. Martínez et al. [34] observed that constant high temperatures increased the number of germinated seeds in species such as *S. portulacastrum* and *S. virginicus* in coastal dunes. Therefore, optimal development of vegetation will be dependent on the location of the vegetation within the profile of the dune and their adaptations to extreme conditions, thus reflected in the SAVI values.

The Bare Soil (sand) category was unexpectedly reduced in both the NZ and SZ of the RSBK, suggesting an increase in plant colonization. This tendency may be the result of relatively stable environmental conditions, the elimination of shrubby and mangrove vegetation, and phases of increased precipitation, especially during the northerlies. Most likely, Bare Soil (sand) was replaced by Low-Density Vegetation like herbaceous and creeping species such as *C. rosea*, *I. pes-caprae*, *S. portulacastrum*, *S. virginicus*, and *C. lanceolate*. These species are considered opportunistic, growing even in the absence of other vegetation. Nevertheless, these plants are also necessary for dune formation and stabilization, which is a desirable initial phase of vegetation recovery in disturbed environments. Globally, a decrease in Bare Soil percentage and an increase in vegetation cover and species richness have been observed. In Brazil, Australia, Venezuela, Argentina, Morocco, South Africa, and other regions of Mexico, similar patterns of bare soil (sand) reduction have also been observed [35,64]. This was possible through long-term analysis as a result of different environmental drivers, including shifts in precipitation, temperature, CO_2_, and the weakening of wind currents from 5% to 15% at lower latitudes [35,64].

## 4. Materials and Methods

### 4.1. Study Area

**Figure 9:**
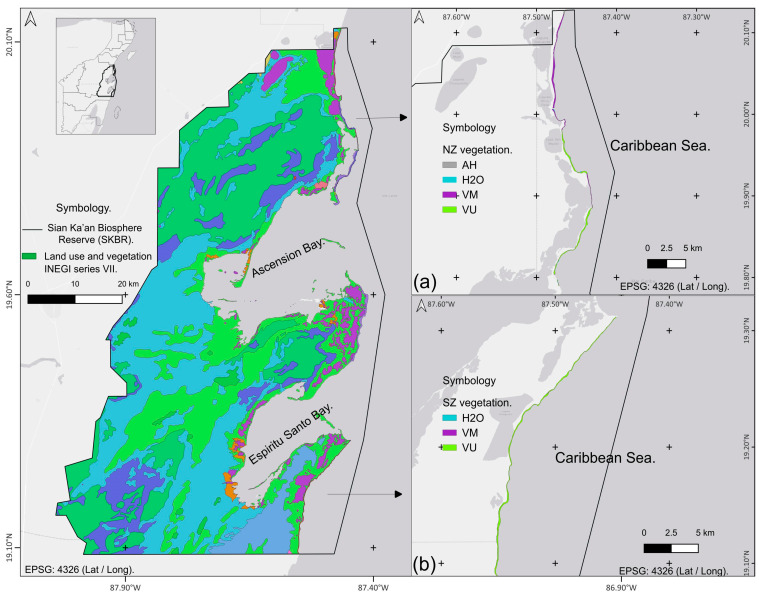
Map showing the geographic position of the Sian Ka’an Biosphere Reserve within the Mexican Caribbean. The reserve is divided into a (**a**) northern zone and a (**b**) southern zone. Colored zones correspond to the land use classification by the Mexican National Institute of Statistics and Geography (INEGI). Only those classification categories relevant to the study area were used. Abbreviations are as follows: AH: human settlement; H2O: water; VM: mangrove vegetation; VU: coastal dune vegetation. Other land use and vegetation categories can be accessed through https://www.inegi.org.mx/app/biblioteca/ficha.html?upc=889463842781 (accessed on 29 April 2024).

The Sian Ka’an Biosphere Reserve (SKBR) is located (Figure 9) to the southeast of the state of Quintana Roo, Mexico (WGS-84) (20.10° N; −86.90° W). This reserve lies within the coasts of the Caribbean Sea, with a beach front of ~80km, characterized by a frontal type dune in different stages of development. Perpendicular to the coastline, dunes extend 100–200 m wide, although only approximately 1600 ha of dune vegetation has been estimated [41]. According to the Köppen climate classification, the region has a tropical savanna climate (Aw) with an average monthly and annual temperature of 22 °C and 26.5 °C, respectively. Average precipitation ranges from 840 to 1550 mm year^−1^ [69], with three well-defined seasons. The dry season extends from March to May, the rainy season from June to October, and the northerlies from November to February [17,70,71]. Dry and rainy seasons are associated with the relative position of the Intertropical Convergence Zone on the American Continent [72]. The northern position of this zone is associated with an increase in humidity budget, whereas the southern position is more associated with dryness [17]. Northerlies are mainly associated with the movement of polar air masses from the northern hemisphere, producing temperature drops and an increase in regional humidity. This season also overlaps with the hurricane season.

The SKBR can be divided into two distinct zones based on their observed anthropogenic impacts (tourist activities, infrastructure, and human settlements) and state of conservation. The surface area of the northern zone (NZ) is ~476,911 ha (Figure 9a), with relatively abundant touristic infrastructure and activities [45,73]. The southern zone (SZ) has a surface area of ~225,468 ha (Figure 9b), with little infrastructure development and tourist arrivals. As a result, this has led to the privatization and segmentation of land into parcels, thus promoting land use change activities [24,25,74].

### 4.2. Estimation of Biannual and Annual SAVI and NDVI Patterns, Using Google Earth Engine

Biannual and annual SAVI and NDVI patterns were estimated using available surface reflectance data in Landsat 7 Level 2, Collection 2, and Tier 1 in Google Earth Engine (GEE). During this process, the approximate incidence of 957 images was verified. All scenes were corrected using the LEDAPS algorithm, version 3.4.048. The SR_B3 and SR_B4 bands were multiplied by a 0.0000275 scale factor and −0.2 compensation factor [75]. Additionally, cloud masking was used to improve the images. Bit 3: Cloud, Bit 4: Cloud Shadow, and Bits 8–9: Cloud Confidence from the Quality Assessment (QA) Pixel Bitmask band were used to evaluate the quality of the pixels [75]. Using the CFMASK algorithm, clouds and cloud shadows were suppressed. The QA band was validated using Bits 8–9 as confidence levels, as this reflects a more realistic measure of the cloud extensions present in the images [75]. Data on maximum temperature (°C), minimum temperature (°C), and precipitation (mm) were obtained from the collection of monthly climate and climatic water balance for global land surfaces (TerraClimate; Tables S1 and S2). The maximum and minimum temperature bands were multiplied by a scale factor of 0.1 for conversion to °C [76]. Equations (1) and (2) were structured in GEE for the estimation of SAVI and NDVI:

SAVI = ((Nir − Red))/((Nir + Red + L)) × (1 + L)(1)

NDVI = ((Nir − Red))/((Nir + Red))(2)

where Nir and Red correspond to the near infrared and red bands (reflectance), respectively. The ground adjustment factor (L) was assigned a constant value of 0.5.

Monthly values for the 2011–2020 period were extracted from the SAVI and NDVI resulting bands. For statistical analysis, biannual layers were constructed, and median values were used, since these are less affected by outliers and/or areas with no data. Five raster layers of SAVI and NDVI were generated at a biannual timeframe. From the biannual and annual layers, the following metrics were estimated: biannual spatial distribution, mosaic density, percentages, and differences in vegetation cover. These results were subsequently represented in QGIS software version 3.28.1 [77] and OriginPro version 9.8.0.200 [78] for visualization.

**Figure 10:**
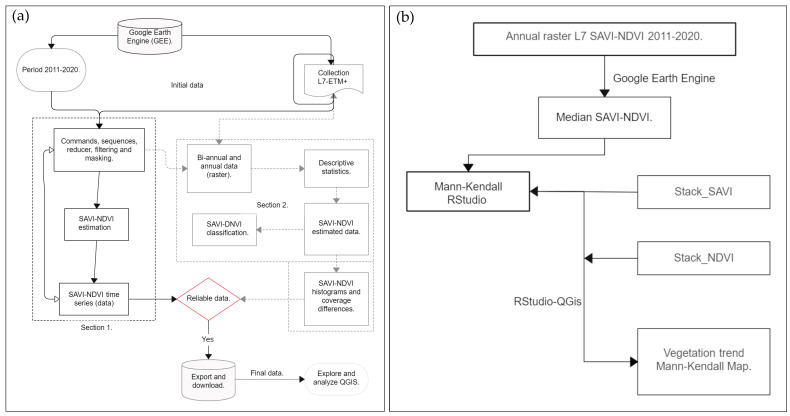
Workflow for the yearly estimation of (**a**) SAVI-NDVI during the 2011–2020 period and (**b**) Mann–Kendall trend for vegetation density change in the Sian Ka’an Biosphere Reserve.

For the comparison of these metrics between zones by timeframe and climatic seasons, SAVI and NDVI data were arranged, respectively. We consider the “northerlies, rainy, and dry” seasons as the three climatic seasons in this work. First, the Kolmogorov–Smirnov (K-S) test was performed, using monthly values for every year extracted from SAVI-NDVI analysis [79]. The Mann–Whitney U test (UMW) was used to estimate significant differences in the density of vegetation cover between indices [80]. Monthly values were arranged by season and analyzed for homogeneity with the K-S test. Subsequently, the Kruskal–Wallis (K-W) test with a 95% confidence level was used to determine significant differences by season. Bonferroni multiple-comparisons correction test was used as a post hoc test. A summary of the workflow of this study is summarized in Figure 10a,b.

### 4.3. Trends in Vegetation Cover Density Changes for the Northern and Southern Zone of the SKBR

To estimate vegetation density change, we classified SAVI into categories following Alencar da Silva-Alves et al. [81]; Bozzi-Zeferino et al. [82]; and Ceceña-Sánchez et al. [31]. In this study, five categories with their associated vegetation per stratum were used to describe the vegetation cover in the coastal dune (Table 1).

The Mann–Kendall (MK) test for monotonic trend [83,84] was used to evaluate trend changes in vegetation cover density. This is a robust analysis that does not require data with a normal distribution and is not very sensitive to outliers [85]. We used RStudio version 1.4.1106, Kendall {Kendall} (2.2.1), RStoolbox (0.3.0), ggplot2 (2.0.0.), brick (3.2–21), sp (1.6–0), rgdal (1.3–3), and raster (3.5–21) packages for analysis. Kendall’s rank correlation measures the strength of the monotonic association between the x and y vectors. This Kendall correlation, through tau, can be expressed as tau=S/D; tau can incorporate values from −1 to 1 (Equation (3)), where:

S = Σ{i < j}(sign(x[j] − x[i]) × sign(y[j] − y[i]))(3)

and D = n(n − 1)/2. S is called the score, and D is the denominator and the maximum possible value of S. The trend is statistically significant if the value of *p* (sl) is <0.05. Tau fluctuates from +1 to −1; a positive value indicates a continued upward trend, the opposite occurs when the value is negative, and a neutral value indicates no consistent trend [86]. The hypotheses of the Mann–Kendall test were as follows. H0: There is no trend in the time series. H1: There is a trend in the time series.

To quantitatively determine the loss or gain of vegetation cover at biannual timeframes, we followed Muhsin [87] and Fassnacht et al. [88] for calculating differences in vegetation cover detected in SAVI and additionally expressing the results in percentage cover between biannual raster layers. The differences in the density of vegetation cover were estimated using the following equation:

IVM_d_ = IVM_2_ − IVM_1_ (4)

where IVM_2_ is the period of interest and IVM_1_ is the previous period.

The density of vegetation cover of the five categories was estimated for SAVI in both northern and southern zones using QGIS software version 3.28.1.

Finally, we evaluated the influence of climate variables on the temporal variability of SAVI by year, season, and zone. In PRIMER V6, distance-based multivariate multiple regression (DistLM) was used to evaluate the internal correlation between maximum and minimum temperature and precipitation. Distance-based redundancy analysis (dbRDA) was conducted to order and visualize patterns of relationships between the data. We used the routine available in Primer V6 that considers the Step-Wise model and an adjusted R2 as a validation factor [89,90,91,92,93]. The DJI Mavic 2 RPAS with a 1/2.3-inch zoom, 12-megapixel sensor with 70% side overlap and 75% front overlap at a height of ~120 m, was used to perform exploratory overflights parallel to the coast over different zones of the study areas for documenting the anthropogenic impact on the coastal dune ecosystem.

## 5. Conclusions

The coastal dune vegetation was dynamic in time and space during the last decade. Hydrometeorological events and seasonality had limited influence on the structure and composition of vegetation. However, we must consider that major climatic events did not occur or directly impact the study area during the study period. It was observed that environmental parameters such as maximum and minimum temperature had a more significant influence on vegetation structure and dynamism than precipitation. Furthermore, the most contrasting climatic periods in the region, the rainy and dry seasons, were in fact not significantly different with regards to vegetation cover during the studied decade. The northerly season was more dynamic, likely because it was associated with temperature drops and more sparsely distributed rains. The most drastic changes were observed in the short term, associated with anthropogenic activities such as the removal of vegetation, tourist infrastructure, and land subdivisions that occurred in the coastal areas of the Wider Caribbean region. The high-density vegetation stratum was the most drastically reduced, since touristic development usually removes this type of vegetation to gain shoreline space. In the North Zone of the SKBR, where tourism has been more oriented towards ecotourism, vegetation recovery was detected, likely because in these zones, pristine environments are more appreciated than devastated. Based on the latter, it can be stated that in this study, anthropogenic activities related to tourism showed two facets of coastal vegetation dynamics. Therefore, the application of remote sensing to obtain rapid and efficient spatio-temporal analyses will remain a helpful tool to develop and establish environmental criteria for better decision making in the management and conservation of natural resources in the Mexican Caribbean. Lastly, permanent monitoring will help analyze the conditions of the coastal dune, its associated vegetation, and its relationship with adjacent ecosystems. This study evaluated long-term vegetation dynamics in the Mexican Caribbean for the first time and will thus serve as an essential benchmark to estimate vegetation changes in the near future under more severe climate variability and more drastic impacts of human activity as a result of economic development in the region.

## Figures and Tables

**Table 1:** Proposed thresholds by category for SAVI.

SAVI		Vegetation Strata and Representative Vegetation
Category 1:	Bare Soil and/or Water (−0.77 to 0);	Sand
Category 2:	Low-Density Vegetation (0 to 0.25);	Herbaceous and creeping vegetation: *Canavalia rosea*, *Ipomoea pes-caprae*, *Sesuvium portulacastrum*, *Sporobolus virginicus*, *Cakile lanceolata.*
Category 3:	Medium–Low-Density Vegetation (0.25 to 0.35);	Shrubby: *Suriana maritima*, *Tournefortia gnaphalodes*, *Ernodea littoralis; Scaevola plumieri*, *Lantana involucrata.*
Category 4:	Medium–High-Density Vegetation (0.35 to 0.45);	Shrubby–arboreal: *Thrinax radiata*, *Ernodea littoralis; Scaevola plumieri*, *Cordia sebestena*, *Tournefortia gnaphalodes*, *Cocos nucifera*, *Lantana involucrata.*
Category 5:	High-Density Vegetation (0.45 to 0.66).	Arboreal: *Thrinax radiata*, *Coccoloba uvifera*, *Cordia sebestena*, *Bursera simaruba*, *Metopium brownei and Cocos nucifer*, *Rhizophora mangle*, *Conocarpus erectus.*

## Data Availability

Data are contained within the article and Supplementary Materials. The SAVI-NDVI estimates at biannual and annual periods, comparative raster layers, as well as climate data (Tmx_°C, Tmn_°C and Prep_mm), and the calculations for the Kendall trend test (R Studio routine) are available at https://github.com/demostenesmx?tab=repositories (accessed on 29 April 2024).

## Supplementary Materials

The following supporting information can be downloaded at: https://www.mdpi.com/article/10.3390/plants13131734/s1, Table S1. Climatic variables of the northern zone of the Sian Ka’an Biosphere Reserve in the Mexican Caribbean. Table S2. Climatic variables of the southern zone of the Sian Ka’an Biosphere Reserve in the Mexican Caribbean.
