# Supplementary material for: Coastal Dune Vegetation Dynamism and Anthropogenic-Induced Transitions in the Mexican Caribbean during the Last Decade
Source: Plants (Basel). 2024 Jun 23;13(13):1734. doi: 10.3390/plants13131734 (PMC11243678; doi:10.3390/plants13131734)
Supplement: Supplementary file 1 [file plants-13-01734-s001.zip › plants-3012813-supplementary.pdf]

Supplementary Material.

Table S1. Climatic variables of the northern zone of the Sian Ka'an Biosphere Reserve in the Mexican Caribbean.

Month	Tmn_C°_NZ	Tmx_C°_NZ	Prep_mm_NZ
Jan_2011	18.4	30	23
Feb_2011	18.7	31.6	34
Mar_2011	20.6	32.8	62
Apr_2011	22.4	33.9	1
May_2011	23.5	35.2	39
Jun_2011	24	34.6	204
Jul_2011	24.5	33.9	125
Aug_2011	23.9	34.4	83
Sep_2011	24	33.4	252
Oct_2011	22.4	30.9	379
Nov_2011	20	30.6	79
Dec_2011	19.8	30	39
Jan_2012	19.7	30.2	57
Feb_2012	20.8	31.4	30
Mar_2012	21.3	33.5	27
Apr_2012	21.8	33	108
May _2012	22.8	33.1	232
Jun_2012	23.8	32.8	265
Jul _2012	23.7	33.7	101
Aug _2012	24	34	101
Sep_2012	23.4	33.8	145
Oct_2012	22.5	32.5	219
Nov_2012	18.7	31	40
Dec_2012	19.4	31.7	20
Jan_2013	20.5	30.5	70
Feb_2013	20.2	31.9	42
Mar_2013	18.5	31.1	30
Apr_2013	22.7	33.7	48
May _2013	22.8	33.7	159
Jun_2013	23.7	32.9	210
Jul _2013	23.6	33.3	175
Aug _2013	23.5	33.5	214
Sep_2013	24.2	31.8	242
Oct_2013	23.1	32.3	272
Nov_2013	21.8	30.5	191
Dec_2013	21.3	30.1	100

Jan_2014	18.4	28.6	83
Feb_2014	20.2	32	58
Mar_2014	20.3	32.6	65
Apr_2014	21.8	33	41
May _2014	22.9	32.4	239
Jun_2014	21.8	34.6	94
Jul _2014	23.6	34.5	63
Aug _2014	23.2	35	102
Sep_2014	23.1	32.3	214
Oct_2014	22.1	32.2	453
Nov_2014	19.4	30.4	167
Dec_2014	19	30.3	55
Jan_2015	18.4	30.5	70
Feb_2015	17.4	31	19
Mar_2015	20.3	32.9	31
Apr_2015	22.5	34.1	29
May _2015	22.8	34.5	44
Jun_2015	23.8	32.9	132
Jul _2015	23.9	34.5	31
Aug _2015	23.2	35.3	111
Sep_2015	23.8	34.2	187
Oct_2015	22.9	32.4	194
Nov_2015	22.6	31.8	170
Dec_2015	22.2	31.4	111
Jan_2016	18.6	29.6	40
Feb_2016	17.6	29.3	43
Mar_2016	20.1	32.1	38
Apr_2016	21.3	32.9	21
May _2016	23	34.2	22
Jun_2016	23.8	33.3	129
Jul _2016	24.5	34.5	43
Aug _2016	23.8	34.1	152
Sep_2016	24	33.4	101
Oct_2016	22.8	32.3	82
Nov_2016	20.9	30.9	22
Dec_2016	21	31.3	29
Jan_2017	19.1	30.1	14
Feb_2017	19.9	31.7	10
Mar_2017	19.6	31.6	10
Apr_2017	21.2	32.7	35
May _2017	22.8	34	49
Jun_2017	23.7	33.3	162
Jul _2017	23.9	33.9	101

Aug _2017	24	34.3	143
Sep_2017	24.2	33.5	177
Oct_2017	22.7	32.2	227
Nov_2017	20.7	30.7	50
Dec_2017	19.9	30.2	24
Jan_2018	18.2	27.8	109
Feb_2018	19.9	31.3	34
Mar_2018	19.4	31.9	55
Apr_2018	20.9	32.8	112
May _2018	22.5	32.4	142
Jun_2018	23.7	32.7	174
Jul _2018	23.3	34.4	79
Aug _2018	22.9	33.6	135
Sep_2018	23.7	33.1	158
Oct_2018	23	32.5	240
Nov_2018	20.9	31.5	116
Dec_2018	19.7	30.1	42
Jan_2019	18.5	29.5	68
Feb_2019	19.9	31.6	38
Mar_2019	20	31.9	25
Apr_2019	21.2	32.7	64
May _2019	22.9	34	54
Jun_2019	24.4	33.9	92
Jul _2019	24.2	34.2	32
Aug _2019	24.1	34.4	83
Sep_2019	24.4	33.8	112
Oct_2019	23.7	33.2	175
Nov_2019	21.5	31.4	93
Dec_2019	20	30.4	47
Jan_2020	19.6	30.7	46
Feb_2020	19.8	31.5	36
Mar_2020	20.6	32.5	19
Apr_2020	22.3	33.8	9
May _2020	23	34	158
Jun_2020	24	33.4	398
Jul _2020	24.1	34.1	57
Aug _2020	23.9	34.2	99
Sep_2020	24.1	33.6	144
Oct_2020	23	32.3	365
Nov_2020	21	31.2	217
Dec_2020	19.3	29.7	80

Table S2. Climatic variables of the southern zone of the Sian Ka'an Biosphere Reserve in the Mexican Caribbean.

Month	Tmn_C°_SZ	Tmx_C°_SZ	Prep_mm_SZ
Jan_2011	19.2	30	28
Feb_2011	19.4	31.2	42
Mar_2011	21.2	32.3	73
Apr_2011	23	33.1	1
May_2011	23.9	34.3	38
Jun_2011	24.2	33.8	223
Jul_2011	24.8	33.1	141
Aug_2011	24.4	33.9	81
Sep_2011	24.3	33.1	264
Oct_2011	22.7	30.6	424
Nov_2011	20.5	30.4	94
Dec_2011	20.3	29.6	50
Jan_2012	20.5	29.9	66
Feb_2012	21.5	30.7	30
Mar_2012	22	32.8	35
Apr_2012	22.6	32.3	112
May _2012	23.3	32.2	254
Jun_2012	24.1	32.1	244
Jul _2012	24.2	33.1	120
Aug _2012	24.4	33.4	103
Sep_2012	23.7	33.5	131
Oct_2012	22.7	32.1	235
Nov_2012	19.3	31	59
Dec_2012	19.9	31.4	27
Jan_2013	21.1	30	91
Feb_2013	20.9	31.3	53
Mar_2013	19.3	30.9	27
Apr_2013	23.4	32.9	43
May _2013	23.5	32.9	178
Jun_2013	24.1	32.2	183
Jul _2013	24.1	32.7	232
Aug _2013	23.9	32.9	238
Sep_2013	24.6	31.3	294
Oct_2013	23.4	32	288
Nov_2013	22.3	30.2	273

Dec_2013	21.6	29.5	125
Jan_2014	19.1	28.3	78
Feb_2014	20.9	31.5	55
Mar_2014	21.2	32.1	75
Apr_2014	22.6	32.3	34
May _2014	23.5	31.5	248
Jun_2014	22.2	33.9	84
Jul _2014	24	33.9	69
Aug _2014	23.6	34.5	95
Sep_2014	23.4	31.9	263
Oct_2014	22.4	31.9	437
Nov_2014	19.9	30.3	208
Dec_2014	19.5	30.1	63
Jan_2015	19.1	30.4	105
Feb_2015	18.2	30.8	15
Mar_2015	21	32.4	37
Apr_2015	23.2	33.4	36
May _2015	23.3	33.7	37
Jun_2015	24.2	32.1	161
Jul _2015	24.3	33.9	24
Aug _2015	23.7	34.8	95
Sep_2015	24.1	33.9	195
Oct_2015	23.1	32.1	226
Nov_2015	23.1	31.6	186
Dec_2015	22.8	31	142
Jan_2016	19.5	29.5	38
Feb_2016	18.5	29	50
Mar_2016	21	31.4	52
Apr_2016	22.2	32.2	18
May _2016	23.5	33.2	22
Jun_2016	24.2	32.6	142
Jul _2016	24.8	33.7	54
Aug _2016	24.3	33.5	177
Sep_2016	24.5	33.1	109
Oct_2016	23.3	32.1	104
Nov_2016	21.4	30.5	55
Dec_2016	21.3	30.8	41
Jan_2017	19.8	29.7	15
Feb_2017	20.3	30.7	12
Mar_2017	20.3	30.8	12
Apr_2017	21.9	31.9	39
May _2017	23.5	33.1	48
Jun_2017	24.1	32.5	182

Jul _2017	24.5	33.3	99
Aug _2017	24.5	33.7	169
Sep_2017	24.7	33.3	182
Oct_2017	23.1	31.8	271
Nov_2017	21.4	30.6	92
Dec_2017	20.4	29.9	38
Jan_2018	19	27.6	153
Feb_2018	20.6	30.7	44
Mar_2018	20.4	31.5	70
Apr_2018	21.6	32	137
May _2018	23.2	31.7	134
Jun_2018	24.2	31.9	211
Jul _2018	23.8	33.7	85
Aug _2018	23.5	33	152
Sep_2018	24.2	32.8	172
Oct_2018	23.2	32.1	267
Nov_2018	21.4	31.2	179
Dec_2018	20.3	29.8	48
Jan_2019	19.3	29.2	85
Feb_2019	20.7	31.1	26
Mar_2019	21	31.4	19
Apr_2019	22	32	107
May _2019	23.5	33.1	56
Jun_2019	24.8	33.3	95
Jul _2019	24.9	33.6	34
Aug _2019	24.9	34	72
Sep_2019	24.7	33.3	117
Oct_2019	24	32.8	192
Nov_2019	21.9	31.1	100
Dec_2019	20.6	30.1	67
Jan_2020	20.3	30.4	54
Feb_2020	20.7	31	31
Mar_2020	21.4	31.9	19
Apr_2020	23.2	33.2	6
May _2020	23.7	33.2	180
Jun_2020	24.5	32.7	302
Jul _2020	24.8	33.4	56
Aug _2020	24.5	33.7	94
Sep_2020	24.6	33.3	163
Oct_2020	23.3	32.1	262
Nov_2020	21.6	31	334
Dec_2020	20	29.5	73
